# Jaceidin Inhibits Proliferation and Promotes Apoptosis in Oral Squamous Cell Carcinoma Cells by Regulating Survivin and AKT/ERK Signaling Pathways

**DOI:** 10.3390/ijms27177871

**Published:** 2026-09-03

**Authors:** Ming-Ju Hsieh, Hsin-Yu Ho, Chia-Chieh Lin, Min-Yun Kao, Yu-Sheng Lo, Yi-Ching Chuang, Bharath Kumar Velmurugan, Mu-Kuan Chen

**Affiliations:** 1 Oral Cancer Research Center, Changhua Christian Hospital, Changhua 50006, Taiwan; 2 Graduate Institute of Clinical Medicine, College of Medicine, National Chung Hsing University, Taichung 402202, Taiwan; 3 Graduate Institute of Biomedical Sciences, China Medical University, Taichung 404328, Taiwan; 4 Department of Dental Technology and Materials Science, Central Taiwan University of Science and Technology, Taichung 406, Taiwan; 5 Maitocon Laboratory, Tirupur 641604, India; 6 Department of Otorhinolaryngology, Head and Neck Surgery, Changhua Christian Hospital, No. 135, Nanxiao St., Changhua 50006, Taiwan

**Keywords:** OSCC, jaceidin, cell viability, apoptosis, MAPK

## Abstract

Oral squamous cell carcinoma (OSCC) remains a clinically challenging malignancy because of recurrence, treatment resistance, and limited therapeutic efficacy in advanced disease. This study investigated the anticancer effects and molecular mechanisms of jaceidin (JAC), a naturally occurring flavonoid derivative, in human OSCC cells. SCC-1 and SCC-47 cells were treated with JAC, and cell viability, colony formation, cell cycle distribution, apoptosis, and apoptosis-related signaling pathways were examined. JAC reduced OSCC cell viability and colony formation in a dose- and time-dependent manner. It also induced G2/M cell cycle accumulation and decreased the expression of cyclin D1, cyclin E1, phosphorylated cdc2, and CDK2/4/6. Apoptosis analyses showed that JAC promoted caspase-dependent apoptotic signaling, as evidenced by increased cleavage of caspase-3, caspase-8, caspase-9, and PARP. JAC modulated death receptor-associated signaling, characterized by increased Fas, TRADD, and DR5 expression and caspase-8 cleavage, while the decoy receptors DcR2 and DcR3 were also upregulated. JAC also promoted mitochondrial apoptotic signaling by increasing Bax, Bak, and Bim expression while decreasing Mcl-1. In addition, JAC attenuated AKT and ERK1/2 phosphorylation, and pharmacological inhibition with LY294002 or U0126 further enhanced JAC-induced apoptotic signaling. Furthermore, JAC reduced survivin expression and attenuated survivin-mediated apoptotic resistance. These findings suggest that JAC suppresses OSCC cell survival in association with modulation of AKT/ERK–survivin signaling and caspase-dependent apoptosis.

## 1. Introduction

Oral squamous cell carcinoma (OSCC) ranks as the seventh most common cancer around the world [1]. The Global Cancer Observatory expects that by 2040, oral cancer incidence will increase by ~40%, with a simultaneous increase in deaths [2]. Alcohol exposure, betel chewing, cigarette smoking, and human papillomavirus infection have been associated with oral cancer occurrence [3]. Current treatment options for OSCC include surgery, radiotherapy, chemotherapy or combination treatment [4]. In spite of the various treatments that have been used over the years, the OSCC overall survival (OS) rate remains at 50% [5,6]. Therefore, it is essential to develop new treatment methods to improve therapeutic efficacy in OSCC.

The MAPK signaling network includes the ERK branch (ERK1/2 and ERK5), as well as the p38 and JNK subfamilies [7,8]. These serine/threonine kinases transmit extracellular cues to intracellular effectors and thereby regulate diverse cellular programs, including proliferation, differentiation, apoptosis, angiogenesis, and metastatic behavior [9]. ERK activation by growth factors suppresses pro-apoptotic proteins and enhances anti-apoptotic protein expression [10]. ERK/MAPK and PI3K/Akt pathways are key signaling cascades that control various cellular functions [11]. The PI3K/AKT pathway plays an essential role in oral cancer progression, and targeting the AKT signaling pathway is an important strategy to treat oral cancer [12].

Jaceidin (5,7,4′-trihydroxy-3,6,3′-trimethoxyflavone) is a naturally occurring polymethoxylated flavonoid isolated from Chamomilla recutita. Structurally, jaceidin contains multiple methoxy and hydroxyl groups, which may contribute to its physicochemical stability and biological activity. Flavonoids are widely recognized for their diverse pharmacological properties, including anti-inflammatory, antioxidant, and anticancer effects. Consistent with these observations, previous studies have demonstrated the anticancer activity of jaceidin in several cancer models. Jaceidin exhibited cytotoxic activity against MCF-7 breast cancer and HepG2 liver cancer cells, with substantially lower cytotoxicity toward the non-malignant HFB-4 cell line, while doxorubicin was included as a positive anticancer control in the same study. Jaceidin has also demonstrated antitumor activity in an Ehrlich ascites carcinoma mouse model and anticancer effects in other malignant cell models. These previous findings provide experimental evidence supporting the anticancer biological activity of jaceidin. However, its effects and underlying mechanisms in oral cancer remain unclear. Therefore, the present study aimed to investigate the effects of jaceidin on cell proliferation and apoptosis in SCC-1 and SCC-47 oral cancer cells and to examine the associated molecular signaling pathways [13,14]. In addition, in silico modeling studies have suggested that jaceidin may preferentially target vascular endothelial growth factor (VEGF) signaling through strong predicted binding to VEGF-associated molecules [14]. However, the potential role of jaceidin in oral cancer has not yet been clarified. Therefore, the present study aimed to investigate the effects of jaceidin on tumor growth inhibition and apoptosis induction in SCC-1 and SCC-47 oral cancer cells. Furthermore, we explored whether jaceidin induces apoptosis through modulation of the MAPK signaling pathway.

## 2. Results

### 2.1. Jaceidin Decreased Oral Cancer Growth

**Figure 1:**
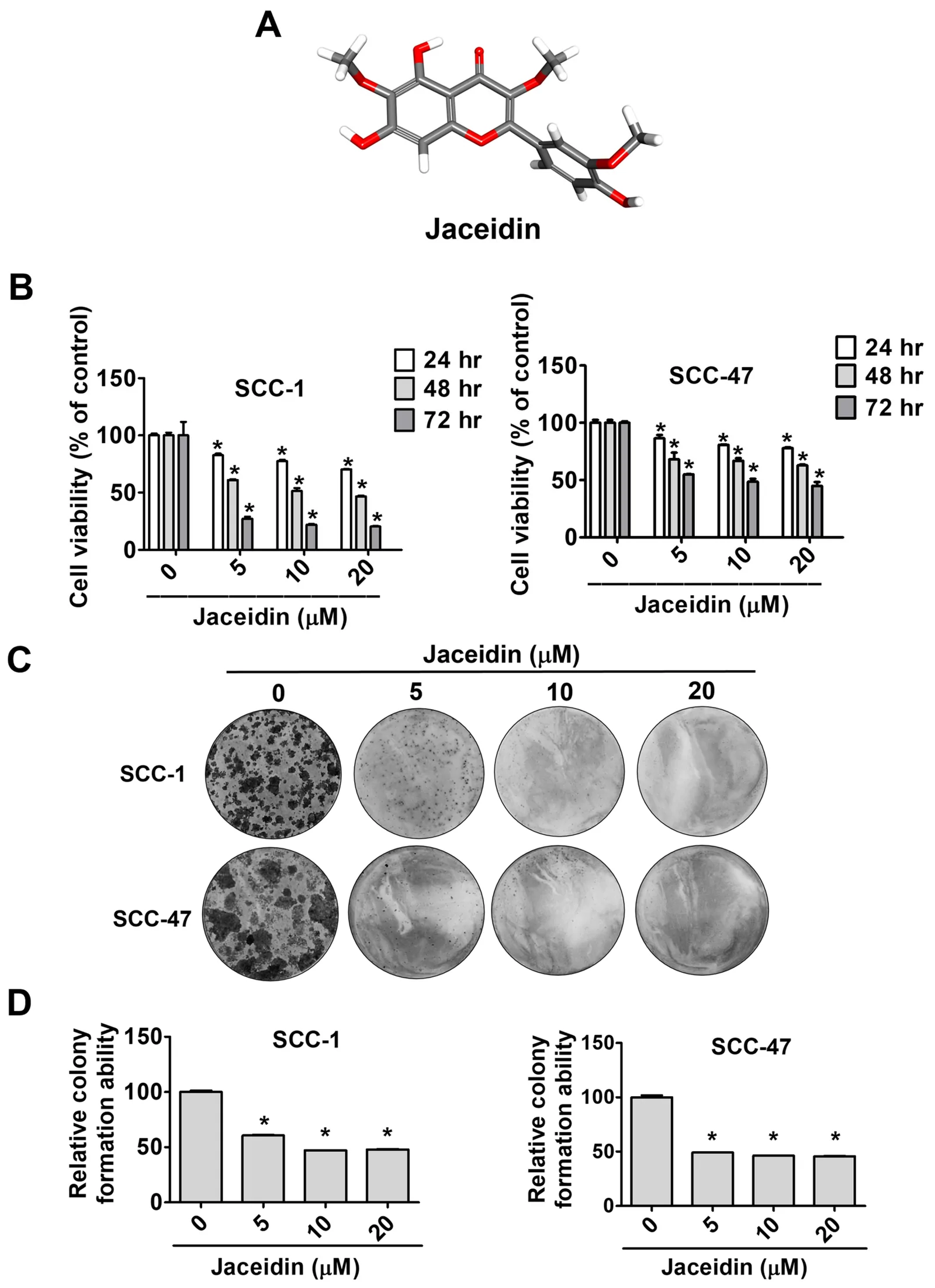
Jaceidin suppresses growth of oral cancer cells. (**A**) Chemical structure of jaceidin. (**B**) SCC-1 and SCC-47 cells were treated with jaceidin (0, 5, 10, or 20 µM) for 24, 48, and 72 h, and cell viability was assessed. (**C**) Representative images of colony formation after exposure to jaceidin, followed by culture for 10 days. (**D**) Colonies were quantified under a stereomicroscope. Data are shown as mean ± SD from three independent experiments (*n* = 3). * *p* < 0.05 versus control.

To examine the effect of jaceidin (Figure 1A) on oral cancer cells, cell viability and colony formation assays were performed. SCC-1 and SCC-47 cells were treated with jaceidin (0, 5, 10, and 20 µM) for 24, 48, and 72 h, and cell viability (Figure 1B) and proliferation were assessed. Jaceidin significantly reduced cell viability. Cells were treated with jaceidin and then cultured for 10 days to evaluate colony formation (Figure 1C,D). There is a noticeable reduction in both the number and size of colonies for SCC-1 and SCC-47 cells in a concentration-dependent manner. These results suggest that jaceidin successfully inhibits the proliferation of different OSCC cell lines.

### 2.2. Jaceidin Effect on Cell Cycle and Its Protein Expression

**Figure 2:**
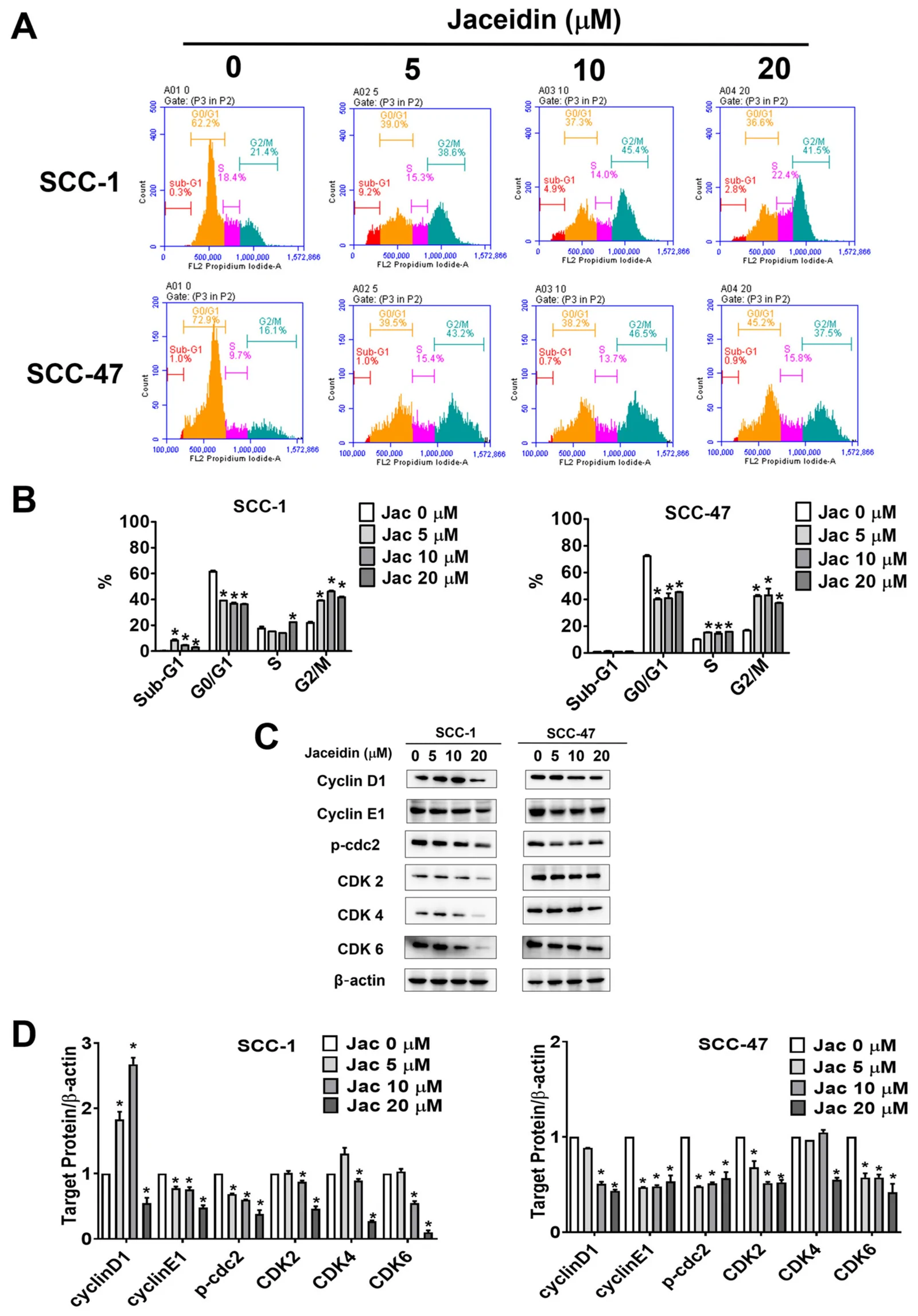
Jaceidin induces G2/M cell cycle arrest in SCC-1 and SCC-47 cells. (**A**) Representative flow cytometry histograms showing cell cycle profiles after jaceidin treatment for 24 h. (**B**) Quantification of cell populations in sub-G1, G0/G1, S, and G2/M phases. (**C**) Immunoblotting of cell cycle regulatory proteins (cyclin D1, cyclin E1, p-cdc2, CDK2, CDK4, and CDK6), with β-actin used as the loading control. (**D**) Densitometric analysis of protein levels. Data are presented as mean ± SD from three independent experiments (*n* = 3). * *p* < 0.05 versus control.

Cell cycle analysis was then conducted on oral cancer cells. Jaceidin treatment significantly increased the G2/M phase in both SCC-1 and SCC-47 cells compared with untreated cells (Figure 2A,B). We further analyzed cell cycle protein expression, including cyclin D1, E1, p-cdc2, CDK2, CDK4, and CDK6. Jaceidin dose-dependently decreased cyclin D1, cyclin E1, p-cdc2, CDK2, CDK4 and CDK6 expression compared to control cells (Figure 2C,D). In this study, jaceidin caused cell cycle arrest in both oral cancer cell lines in association with decreased cell cycle protein expression.

### 2.3. Jaceidin-Mediated Cancer Cell Death

**Figure 3:**
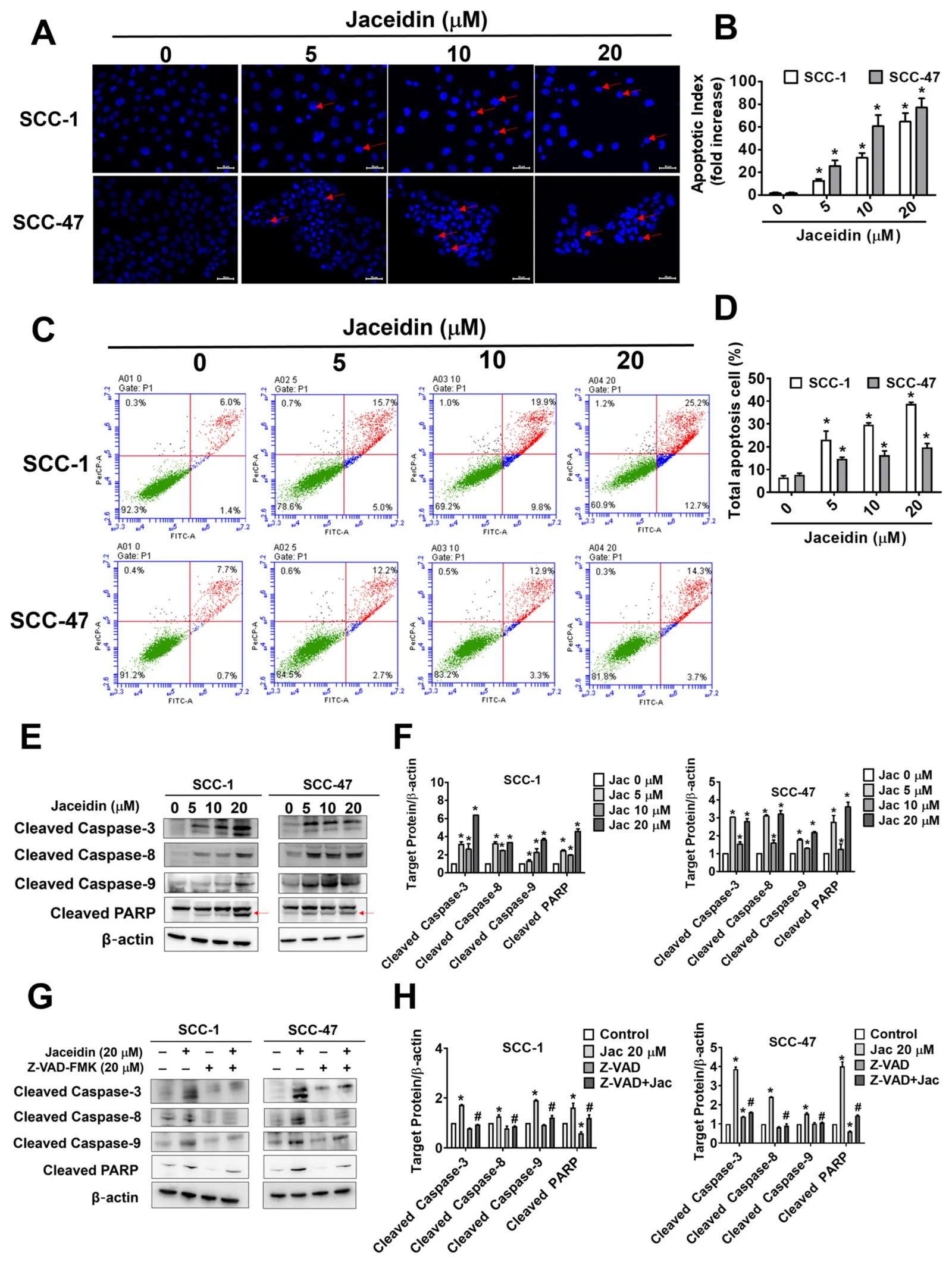
Jaceidin activates caspase-dependent apoptosis in OSCC cells. SCC-1 and SCC-47 cells were exposed to jaceidin for 24 h. (**A**) DAPI staining shows nuclear condensation/fragmentation (scale bar, 50 µm; arrows indicate condensed nuclei). (**B**) Quantification of DAPI-positive apoptotic nuclei. (**C**) Annexin V/propidium iodide (PI) staining followed by flow cytometric analysis to assess apoptosis. (**D**) Percentage of apoptotic populations. (**E**) Immunoblotting of cleaved caspase-3, -8, and -9 and cleaved PARP, with β-actin as the loading control. (The red arrows indicate the positions of the target protein bands). (**F**) Densitometric quantification of apoptotic markers. (**G**) Cells were pretreated with the pan-caspase inhibitor Z-VAD-FMK for 1 h prior to jaceidin exposure, followed by immunoblot analysis. (**H**) Quantification of the corresponding protein levels. Data are presented as mean ± SD from three independent experiments (*n* = 3). * *p* < 0.05 versus control. # *p* < 0.05 versus jaceidin alone.

To study whether jaceidin induces apoptosis in oral cancer cells, cells were treated with jaceidin for 24 h. As shown in Figure 3A, jaceidin treatment induced apoptotic nuclear condensation and fragmentation with respect to dosage. Quantitative analysis showed a significant increase in the apoptotic index in both cell lines (Figure 3B). We then analyzed apoptosis induction by Annexin V/PI staining. Jaceidin treatment increased apoptosis of oral cancer cells in a dose-dependent manner (Figure 3C,D). We then determined the altered expression levels of apoptosis proteins by Western blotting. Jaceidin treatment increased cleaved caspases-3, -8, and -9, and cleaved PARP levels in oral cancer cells. Additionally, to evaluate the role of caspase-3/9 in jaceidin-induced apoptosis in SCC-1 and SCC-47 cells, an experiment was carried out after dividing the cells into the control group, pan-caspase inhibitor (Z-VAD-FMK) treatment group, jaceidin treatment group, and jaceidin combined pretreatment 1 h pan-caspase inhibitor group. The jaceidin treatment group showed results similar to previous findings (Figure 3E); in the pan-caspase inhibitor group, the protein levels of caspase-3, -8, -9, and cleaved PARP decreased. Whereas in the combined treatment group, decreased protein expression by the caspase inhibitor was reversed upon jaceidin treatment.

### 2.4. Jaceidin Induces Apoptosis by Extrinsic and Intrinsic Apoptotic Signaling Pathways

**Figure 4:**
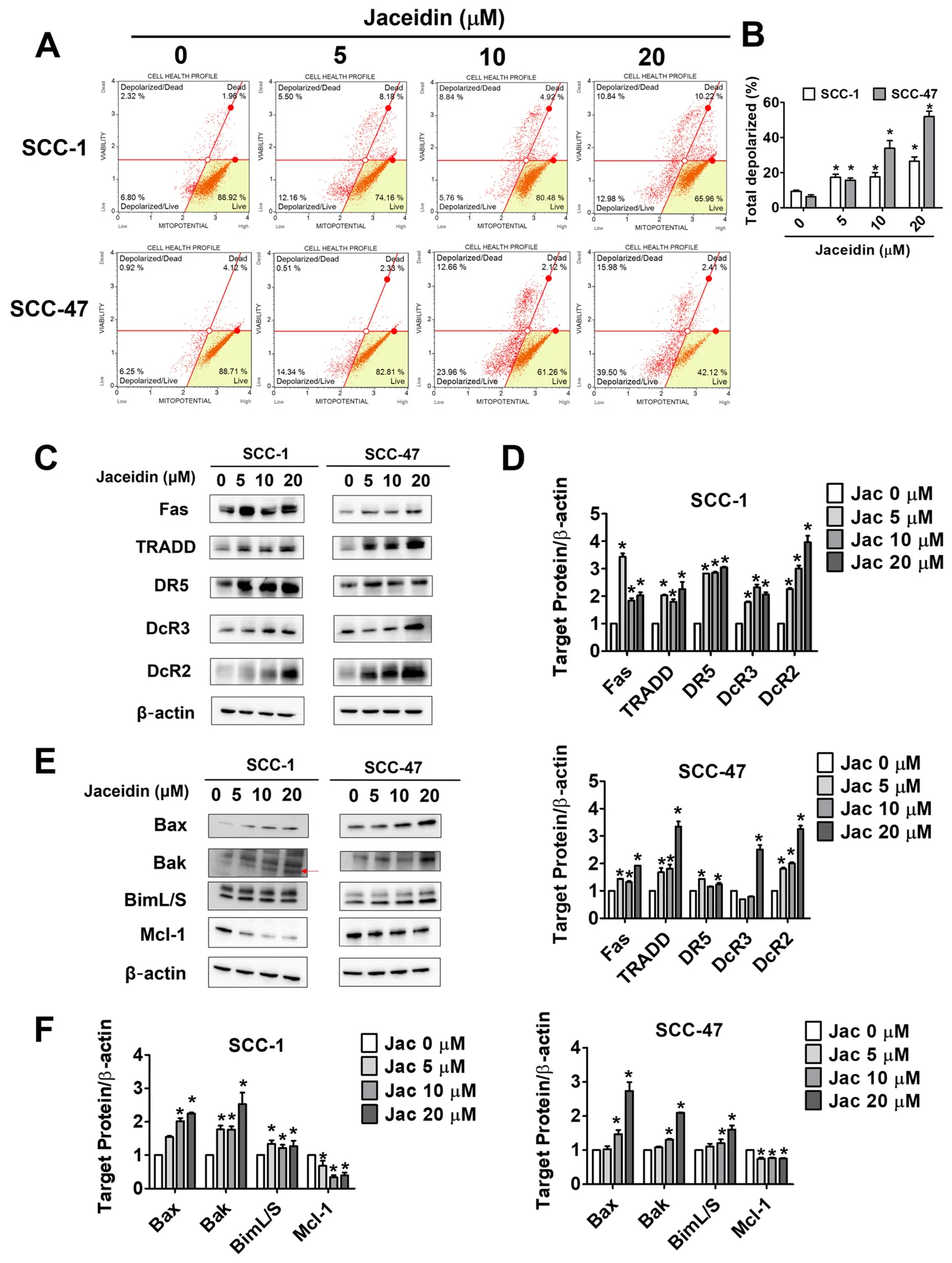
Jaceidin modulates death receptor-associated proteins and mitochondrial apoptotic signaling in OSCC cells. SCC-1 and SCC-47 cells were exposed to jaceidin for 24 h. (**A**) Representative flow cytometry plots showing mitochondrial membrane depolarization in SCC-1 and SCC-47 cells after jaceidin exposure. (**B**) Quantification of depolarized cell populations. (**C**) Immunoblot analysis of extrinsic pathway-related proteins (TRADD, Fas, DR5, DcR2, and DcR3), with β-actin serving as the loading control. (**D**) Densitometric quantification of intrinsic pathway-associated proteins in SCC-1 and SCC-47 cells. (**E**) Immunoblot analysis of mitochondrial apoptosis regulators (Bax, Bak, BimL/S, and Mcl-1), with β-actin as the loading control. (The red arrows indicate the positions of the target protein bands). (**F**) Densitometric quantification of extrinsic pathway-associated proteins in SCC-1 and SCC-47 cells. Data are shown as mean ± SD from three independent experiments (*n* = 3). * *p* < 0.05 versus control.

Jaceidin-induced mitochondrial membrane potential (MMP) change was determined by a 7-AAD-based flow cytometry assay. As shown in Figure 4A,B, jaceidin treatment dose-dependently increased mitochondrial membrane potential in both SCC-1 and SCC-47 cells. To further evaluate the involvement of death- and decoy receptor-mediated signaling, Fas, TRADD, DR5, DcR2 and DcR3 were examined [15,16]. As shown in Figure 4C,D, jaceidin treatment increased the expression of the death receptor-associated proteins Fas, TRADD, and DR5 in both SCC-1 and SCC-47 cells. Interestingly, expression of the decoy receptors DcR2 and DcR3 also increased following jaceidin treatment. Together with the observed cleavage of caspase-8, these findings indicate that jaceidin alters multiple components associated with death receptor signaling; however, the functional contribution of individual death receptors to jaceidin-induced apoptosis remains to be established. Next, we sought to understand the mechanism of jaceidin-induced apoptosis via the intrinsic mitochondrial pathway. Cells were treated with jaceidin and then analyzed for several members of the Bcl-2 family (Bax, Bak, BimL/S and Mcl-1). We found the protein expressions of Bax, Bak and Bim were all upregulated in jaceidin-treated cells, whereas expression of the anti-apoptotic protein Mcl-1 decreased in a dose-dependent manner (Figure 4E,F).

### 2.5. Jaceidin-Induced Apoptosis by MAPK Pathway

**Figure 5:**
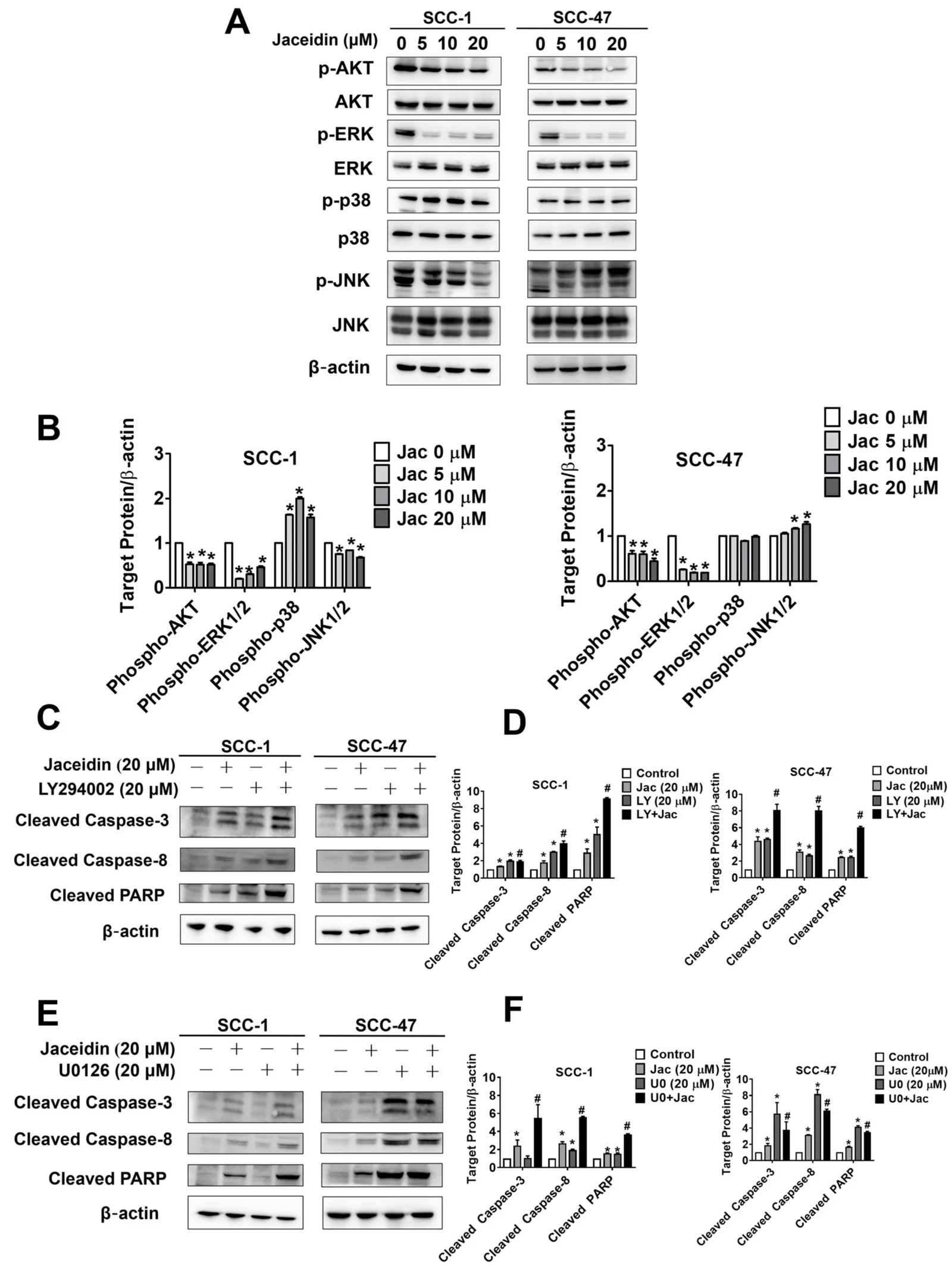
Effects of jaceidin on AKT/MAPK signaling and apoptotic responses in OSCC cells. SCC-1 and SCC-47 cells were exposed to jaceidin for 24 h. (**A**) Immunoblot analysis of p-AKT, p-ERK1/2, p-p38, and p-JNK1/2 following jaceidin treatment, with β-actin used as the loading control. (**B**) Densitometric quantification of phosphorylated kinase levels. (**C**) Cells were pre-incubated with the AKT inhibitor LY294002 for 1 h prior to jaceidin exposure (24 h), and apoptotic markers were examined by immunoblotting (β-actin, loading control). (**D**) Quantification of cleaved caspase-associated proteins and PARP cleavage. (**E**) Cells were pretreated with the ERK pathway inhibitor U0126 for 1 h and then treated with jaceidin for 24 h, followed by immunoblot analysis (β-actin, loading control). (**F**) Quantification of cleaved caspase-related proteins and PARP cleavage. Data are presented as mean ± SD from three independent experiments (*n* = 3). * *p* < 0.05 versus control; # *p* < 0.05 versus jaceidin alone.

To examine the signaling mechanisms underlying jaceidin-induced apoptosis, we analyzed the role of MAPK and AKT pathways in SCC-1 and SCC-47 cells. As shown in Figure 5A, jaceidin treatment markedly decreased the phosphorylation levels of AKT and ERK1/2 in both SCC-1 and SCC-47 cells and increased p38 phosphorylation levels in only SCC-1 cells. No significant increase in p38 phosphorylation was found in SCC-47 cells (Figure 5A–C). Phosphorylated JNK1/2 decreased in SCC-1 cells, whereas it increased in SCC-47 cells. To further assess the role of AKT signaling, cells were pretreated with the AKT inhibitor LY294002 before jaceidin treatment. Combination treatment with LY294002 and jaceidin markedly enhanced the levels of cleaved caspase-3, -8, and cleaved PARP compared with jaceidin alone, showing that AKT inhibition further enhances jaceidin-associated apoptotic signaling (Figure 5D,E). Similarly, pretreatment with the ERK inhibitor (U0126) further augmented jaceidin-induced activation of apoptotic markers (Figure 5F). These results indicate that jaceidin-induced apoptosis is associated with suppression of AKT and ERK1/2 signaling, while pharmacological inhibition of these pathways further enhances jaceidin-induced apoptotic signaling.

### 2.6. Role of Jaceidin on Survivin Protein Expression

**Figure 6:**
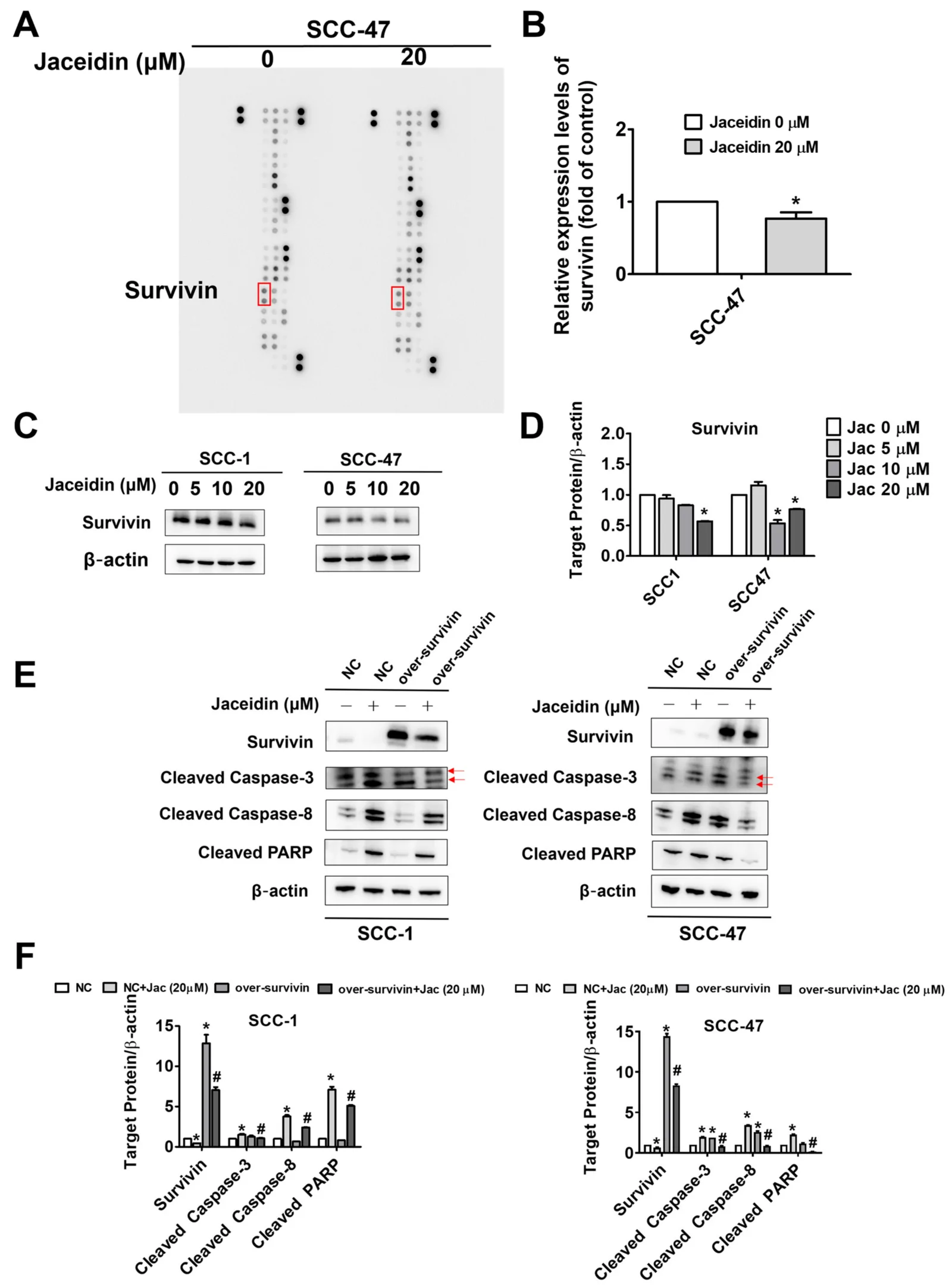
Survivin modulates the apoptotic response to jaceidin in OSCC cells. (**A**) Apoptosis proteome array (Proteome Profiler) showing reduced survivin signal in SCC-47 cells after 24 h of jaceidin exposure. (The red boxes indicate the positions of the target protein). (**B**) Densitometric quantification of survivin from the array. (**C**) Immunoblot validation of survivin downregulation in SCC-1 and SCC-47 cells, with β-actin as the loading control. (**D**) Quantification of survivin protein levels. (**E**) Cells transfected with a survivin overexpression plasmid (over-survivin) were treated with jaceidin, and apoptosis-related markers were assessed by immunoblotting. (The red arrows indicate the positions of the target protein bands). (**F**) Quantification of cleaved caspases and PARP cleavage. Data are shown as mean ± SD from three independent experiments (*n* = 3). * *p* < 0.05 versus control; # *p* < 0.05 versus jaceidin alone.

Based on our previous study [16], in this study we further analyzed apoptosis-related proteins by apoptosis array in jaceidin-treated SCC-47cells. As illustrated in Figure 6A,B, survivin protein levels were markedly decreased after treatment with 20 µM jaceidin. This finding was further validated by immunoblotting in both SCC-1 and SCC-47 cells, where jaceidin produced a concentration-dependent reduction in survivin expression (Figure 6C,D). To determine whether survivin modulates the apoptotic response to jaceidin, survivin was ectopically expressed in oral cancer cells, followed by assessment of apoptotic markers with or without jaceidin exposure. Survivin overexpression attenuated jaceidin-induced activation of caspase-3 and caspase-8 and reduced PARP cleavage (Figure 6E,F). Collectively, these results indicate that downregulation of survivin contributes to jaceidin-mediated activation of caspase signaling and apoptotic execution in OSCC cells.

## 3. Discussion

Oral squamous cell carcinoma (OSCC) is one of the most frequently diagnosed malignancies in Southeast Asia and is commonly associated with an unfavorable prognosis and high mortality rates [17]. Previous studies have demonstrated the anticancer activity of jaceidin in several experimental models. Notably, jaceidin exhibited substantially greater cytotoxicity toward HepG2 and MCF-7 cancer cells than toward the non-malignant HFB-4 cell line, and its activity was evaluated alongside doxorubicin as a positive control [14]. Additional studies have also demonstrated the anticancer activity of jaceidin in other malignant cell models and in vivo [18,19,20]. These findings provide prior experimental support for the anticancer biological activity of jaceidin. However, its biological activity and mechanistic relevance in oral cancer have not been comprehensively examined. Therefore, the present study was designed to investigate the effects of jaceidin in OSCC cells and the associated molecular mechanisms.

Our findings demonstrate that jaceidin markedly suppresses OSCC cell viability and proliferation in a concentration-dependent manner. Apoptotic induction was clearly evidenced by enhanced DAPI-positive nuclear condensation and increased annexin V–positive cell populations in both SCC-1 and SCC-47 cells. The apoptotic cascade is often driven by upstream caspases (caspase-8 and caspase-9) that amplify the signal to caspase-3, thereby promoting PARP cleavage and execution of cell death [21,22]. Consistent with this cascade, jaceidin treatment significantly elevated the levels of cleaved caspase-3, -8, -9, and PARP. To further verify the involvement of caspases in jaceidin-induced apoptosis, a pan-caspase inhibitor was employed. Inhibition of caspase activity substantially reduced the cleavage of caspases and PARP, whereas subsequent jaceidin exposure partially restored these apoptotic markers. These results indicate that activation of the caspase-dependent pathway plays a critical role in mediating jaceidin-induced apoptosis in oral cancer cells.

In addition to apoptosis induction, cell cycle dysregulation represents a key mechanism underlying the anti-cancer activity of numerous chemotherapeutic agents [23,24]. Cell cycle progression is precisely controlled by cyclins and cyclin-dependent kinases (CDKs), and disruption of these regulators often results in growth arrest [25]. In the present study, jaceidin treatment caused a pronounced accumulation of OSCC cells at the G2/M phase. This effect was accompanied by a coordinated reduction in the expression of cyclin D1, cyclin E1, p-cdc2, CDK2, CDK4, and CDK6. Similar regulatory patterns have been reported for other naturally derived compounds that induce G2/M arrest through suppression of CDK–cyclin complexes [26]. Our data suggest that jaceidin interferes with cell cycle progression by targeting multiple regulatory proteins, thereby contributing to its growth-inhibitory effects in OSCC cells.

There are two types of apoptosis pathways, which include extrinsic (Fas or TNF receptors) and intrinsic pathways. The tumor necrosis factor receptor family comprises TNFRI, Fas, DR3, DR4, DR5, and DR6, which are important for regulating apoptotic processes in various physiological systems [27]. The death domain (DD) is essential for transducing apoptotic signals by recruiting adaptor proteins like FADD, TRADD and RIP to activate caspases [28]. Alternatively, death receptor signaling is also controlled by cell surface decoy receptors-DcR1, DcR2 and DcR3. Decoy receptors have a truncated death domain, and they can bind to ligands, but they cannot induce further signals [29]. In the present study, jaceidin increased the expression of Fas, TRADD, and DR5, accompanied by increased caspase-8 cleavage, suggesting an association between jaceidin-induced apoptosis and death receptor-related signaling. However, jaceidin also increased the expression of DcR2 and DcR3. Because these decoy receptors lack functional intracellular death domains and can antagonize death receptor-mediated apoptotic signaling, their upregulation should not be interpreted as evidence of extrinsic pathway activation. Instead, the concomitant induction of decoy receptors may represent a compensatory cellular response to altered death receptor signaling. The biological significance of this response remains unclear. Moreover, because functional blockade or genetic depletion of Fas or DR5 was not performed, the present findings cannot establish a causal requirement for the extrinsic pathway in jaceidin-induced apoptosis. Further studies using receptor-specific neutralizing antibodies or gene-silencing approaches will be required to clarify this mechanism. Intrinsic or mitochondrial apoptosis is mostly regulated by BCL-2 proteins [30]. Upon DISC activation, initiator caspases are activated and can amplify apoptosis by linking to the mitochondrial pathway through Bid cleavage and Bax-mediated mitochondrial dysfunction [31]. Gefitinib promotes apoptosis by engaging the mitochondrial pathway, characterized by increased expression of the proapoptotic Bcl-2 family members Bax and Bim [32,33]. Bax and Bak play a crucial role in facilitating mitochondrial permeabilization [34]. Activator BH3-only molecules (BH3s) such as Bim, Puma and Bid are usually essential initiators to trigger the Bax/Bak-dependent intrinsic apoptosis pathway [35]. Mcl-1 is a member of the Bcl-2 protein family and plays a key role in the dysregulation of programmed cell death [36]. In the present study, jaceidin induced apoptosis by inducing MMP in oral cancer cells. Furthermore, jaceidin induced pro-apoptotic proteins Bak, Bax, Bim and decreased anti-apoptotic proteins Mcl-1, suggesting that jaceidin-induced apoptosis in SCC-1 and SCC-47 cells might occur partly through a mitochondrion-mediated pathway. While the present findings, including mitochondrial membrane depolarization, altered expression of Bcl-2 family proteins, and activation of caspase-9, support the involvement of mitochondria-mediated apoptosis, direct assessment of ROS production and cytochrome c release was not performed. Therefore, future studies will be required to further validate the contribution of mitochondrial signaling events to jaceidin-induced apoptotic cell death in OSCC cells.

Earlier findings indicate that naringenin-induced cell apoptosis occurs via the PI3K/AKT and MAPK signaling pathways in prostate cancer cells [37]. Apoptotic signaling has been shown to involve several MAPK family members, including ERK1/2, JNK, and p38 kinases. We previously reported that Semilicoisoflavone B triggers apoptotic cell death in oral cancer cells by suppressing activation of AKT and MAPK signaling (ERK1/2, p38, and JNK1/2), as reflected by decreased phosphorylation of these kinases [16]. In another study, limocitrin inhibited AKT, ERK1/2 and JNK phosphorylation and upregulated p38 phosphorylation [24]. In this present study, jaceidin treatment decreased AKT and ERK1/2 phosphorylation in SCC-1 and SCC-47 cells, suggesting inhibition of two major pro-survival pathways. Additionally, jaceidin increased p38 expression in SCC-1 cells, whereas this increase was not significant in SCC-47 cells. Interestingly, JNK1/2 phosphorylation decreased in SCC-1 cells but increased in SCC-47 cells following jaceidin exposure. This suggests cell line-dependent differences in signaling responses. Therefore, the observed reduction in p-AKT and p-ERK1/2 levels suggests that jaceidin disrupts essential pro-tumorigenic signaling cascades, thereby shifting the cellular balance toward apoptosis. To further assess the role of AKT signaling, cells were pretreated with the AKT inhibitor LY294002 before jaceidin treatment. Combination treatment with LY294002 and jaceidin markedly enhanced the levels of cleaved caspase-3, cleaved caspase-8, and cleaved PARP compared with jaceidin alone, suggesting that inhibition of AKT potentiates jaceidin-induced apoptosis (Figure 5D,E). Similarly, pretreatment with the ERK inhibitor U0126 further augmented jaceidin-induced activation of apoptotic markers (Figure 5F). We next investigated whether the inhibition of AKT or ERK is essential for apoptosis. AKT inhibitor (LY294002) and ERK inhibitor (U0126) were used to determine whether the inhibition of these two proteins was responsible for apoptosis induction. Pharmacological inhibition of AKT using LY294002 markedly increased jaceidin-induced cleavage of caspase-3, caspase-8, and PARP, indicating that pharmacological AKT inhibition further sensitizes cells to jaceidin-associated apoptotic signaling. Consistently, blockade of ERK1/2 activity with U0126 produced a similar enhancement of apoptotic marker activation. These data indicate that jaceidin treatment is associated with alterations in key pro-survival signaling pathways. Interestingly, differential regulation of MAPK signaling was observed between SCC-1 and SCC-47 cells following jaceidin treatment. While p38 activation was more prominent in SCC-1 cells, distinct JNK1/2 responses were detected between the two cell lines. These differences may reflect intrinsic variations in genetic background, signaling network regulation, and cellular stress-response mechanisms. Previous studies have demonstrated that MAPK signaling can exhibit cell type-specific activation patterns depending on the molecular characteristics of individual cancer cell lines. Importantly, despite these differences in p38 and JNK1/2 modulation, jaceidin consistently reduced cell viability and induced apoptosis in both SCC-1 and SCC-47 cells, suggesting that its pro-apoptotic effects are not solely dependent on a single MAPK signaling branch.

In addition, extensive crosstalk has been reported between the ERK and PI3K/AKT signaling pathways in various cancer types. These pathways cooperatively regulate cell survival, proliferation, and apoptosis through complex feedback and compensatory mechanisms. In the present study, pharmacological inhibition of either ERK or AKT signaling further enhanced jaceidin-induced apoptotic signaling, suggesting that these pathways may contribute to the survival response of OSCC cells under jaceidin treatment. Importantly, additional experiments using lower concentrations of jaceidin showed that AKT and ERK1/2 phosphorylation was reduced even at concentrations that produced minimal effects on cell viability. These findings suggest that alterations in AKT/ERK signaling can occur under minimally cytotoxic conditions and are therefore unlikely to arise exclusively as a secondary consequence of extensive cell death. Nevertheless, the present findings do not establish that AKT/ERK suppression is the direct upstream cause of jaceidin-induced apoptosis. Therefore, the AKT/ERK findings should be interpreted as supporting an association between AKT/ERK modulation and the cellular response to jaceidin rather than as definitive evidence of pathway dependency. The potential crosstalk between the ERK and PI3K/AKT pathways also remains to be elucidated. Further studies employing genetic rescue and early time-course analyses will be required to establish the temporal and causal relationship between AKT/ERK modulation and jaceidin-induced apoptosis.

We previously demonstrated that survivin critically modulates the apoptotic response induced by Semilicoisoflavone B in oral cancer cells, supporting its role as a key molecular target [16]. In our present study, jaceidin treatment inhibited survivin expression in SCC-47 cells, and survivin overexpression decreased caspase-3, -8, and PARP cleavage. However, jaceidin treatment rescued this apoptotic protein expression in survivin-overexpressing cells.

## 4. Materials and Methods

### 4.1. Cell Culture

Human oral squamous cell carcinoma (OSCC) cell lines SCC-1 and SCC-47 were obtained from the Japanese Collection of Research Bioresources (JCRB) Cell Bank (Osaka, Japan). SCC-1 cells were originally derived from a primary oral squamous cell carcinoma of the oral cavity, whereas SCC-47 cells were established from human head and neck squamous cell carcinoma and exhibit distinct biological characteristics compared with other OSCC cell lines. Cells were cultured in Dulbecco’s Modified Eagle Medium (DMEM) supplemented with 10% fetal bovine serum (FBS) and 1% penicillin–streptomycin (all from Gibco, Thermo Fisher Scientific, Waltham, MA, USA) and maintained at 37 °C in a humidified incubator with 5% CO_2_.

### 4.2. Reagents and Chemical Treatment

Jaceidin (purity ≥ 96%) was purchased from ChemFaces (Wuhan, China) [38,39]. Dimethyl sulfoxide (DMSO) was purchased from Sigma-Aldrich (St. Louis, MO, USA). Jaceidin was dissolved in DMSO to prepare a 10 mM stock solution, which was aliquoted and stored at −20 °C until use. For experimental treatments, the stock solution was freshly diluted with the appropriate culture medium to obtain the desired working concentrations. The final concentration of DMSO in the culture medium was maintained below 0.1% (*v*/*v*) in all experiments to minimize solvent-induced cytotoxicity. Cells treated with culture medium containing 0.1% DMSO alone served as the vehicle control. Before each experiment, the diluted jaceidin solutions were gently mixed and added to the cell culture medium at the indicated concentrations for the specified treatment times.

### 4.3. Cell Viability and Colony Formation Assays

Cell viability was determined using the WST-8 colorimetric assay (AAT Bioquest, Sunnyvale, CA, USA). Briefly, SCC-1 and SCC-47 cells were seeded in 96-well plates at a density of approximately 5 × 10^3^ cells per well and allowed to attach overnight. Cells were then treated with the indicated concentrations of jaceidin for 24, 48, and 72 h. After treatment, 10 µL of WST-8 reagent was added to each well containing 100 µL of culture medium, followed by incubation at 37 °C for 1–2 h. The absorbance was measured at 460 nm using a Synergy HTX multimode microplate reader (BioTek Instruments, Winooski, VT, USA), and cell viability was calculated as a percentage relative to the untreated control group. For the colony formation assay, SCC-1 and SCC-47 cells were seeded in 6-well plates at a density of approximately 500–1000 cells per well and allowed to adhere overnight. Cells were exposed to jaceidin for 24 h and subsequently cultured for 10 days to allow colony formation. Colonies were subsequently fixed with 4% paraformaldehyde (Santa Cruz Biotechnology, Dallas, TX, USA) and stained with Giemsa stain (Thermo Fisher Scientific, Waltham, MA, USA). Visible colonies containing more than 50 cells were counted under a light microscope, and representative images were recorded.

### 4.4. Cell Cycle and Apoptosis Analysis

Cell cycle distribution was analyzed by flow cytometry following DNA staining with propidium iodide (PI). Briefly, SCC-1 and SCC-47 cells were treated with the indicated concentrations of jaceidin for the specified time period. Cells were harvested, washed with phosphate-buffered saline (PBS), and fixed in 70% cold ethanol at −20 °C overnight. After fixation, cells were washed with PBS and incubated with RNase A (50 µg/mL) and propidium iodide (50 µg/mL; BD Pharmingen Propidium Iodide Staining Solution, BD Biosciences, San Jose, CA, USA) in the dark at 37 °C for 30 min. The DNA content of stained cells was analyzed using a BD Accuri C6 Plus Personal Flow Cytometer (BD Biosciences, San Jose, CA, USA), and the distribution of cells in the G0/G1, S, and G2/M phases was determined using BD Accuri C6 software; BD Biosciences, San Jose, CA, USA). Apoptotic cell death was evaluated using the BD Pharmingen FITC Annexin V Apoptosis Detection Kit I (BD Biosciences, San Jose, CA, USA). After treatment, cells were collected, washed twice with cold PBS, and resuspended in binding buffer. Cells were then incubated with Annexin V-FITC and PI for 15 min at room temperature in the dark according to the manufacturer’s instructions. The stained cells were immediately analyzed by flow cytometry to quantify the percentages of early apoptotic, late apoptotic, and necrotic cells. In addition, nuclear morphological changes associated with apoptosis were examined by DAPI staining (Santa Cruz Biotechnology, Dallas, TX, USA). Treated cells were fixed with 4% paraformaldehyde, washed with PBS, and stained with DAPI solution (1 µg/mL) for 10 min in the dark. Nuclear morphology was then observed under a fluorescence microscope (Leica Microsystems, Wetzlar, Germany), and apoptotic features such as chromatin condensation and nuclear fragmentation were recorded.

### 4.5. Mitochondrial Membrane Potential Assay

Changes in mitochondrial membrane potential (ΔΨm) were assessed using the Muse MitoPotential Assay Kit (Luminex Corporation, Austin, TX, USA) according to the manufacturer’s instructions. Briefly, SCC-1 and SCC-47 cells were seeded in a culture dish and treated with the indicated concentrations of jaceidin for the specified time period. After treatment, cells were harvested, washed with phosphate-buffered saline (PBS), and resuspended in assay buffer at a density of approximately 1 × 10^5^ cells per sample. The cell suspension was incubated with Muse MitoPotential dye for 20 min at 37 °C in the dark, followed by the addition of 7-aminoactinomycin D (7-AAD) to distinguish dead cells from viable cells. After incubation, samples were immediately analyzed using the Muse Cell Analyzer (Luminex Corporation, Austin, TX, USA). Cells exhibiting mitochondrial depolarization were identified by changes in fluorescence intensity of the MitoPotential dye. The percentages of cells with intact mitochondrial membrane potential and depolarized mitochondria were quantified using the Muse analysis software (version 1.9; Luminex Corporation, Austin, TX, USA) according to the manufacturer’s recommended gating strategy.

### 4.6. Immunoblotting Analysis

Total cellular proteins were extracted using RIPA lysis buffer (Thermo Fisher Scientific, Waltham, MA, USA) supplemented with protease and phosphatase inhibitor cocktails. The protein concentration was determined using the bicinchoninic acid (BCA) protein assay kit (Thermo Fisher Scientific, Waltham, MA, USA) according to the manufacturer’s instructions. Equal amounts of protein (20 μg) were separated by 10–12% SDS–polyacrylamide gel electrophoresis (SDS-PAGE) and subsequently transferred onto polyvinylidene difluoride (PVDF) membranes. After transfer, membranes were blocked with 5% non-fat milk in Tris-buffered saline containing 0.1% Tween-20 (TBST) for 1 h at room temperature and then incubated overnight at 4 °C with primary antibodies against cell cycle regulatory proteins, apoptosis-related proteins, and MAPK/AKT signaling pathway components. After washing with TBST, membranes were incubated with the appropriate horseradish peroxidase (HRP)-conjugated secondary antibodies for 1 h at room temperature. Immunoreactive protein bands were detected using an enhanced chemiluminescence (ECL) detection system and visualized with a ChemiDoc MP Imaging System (Bio-Rad Laboratories, Hercules, CA, USA). Band intensities were quantified by densitometric analysis using ImageJ software (version 1.54g; National Institutes of Health, Bethesda, MD, USA), and β-actin was used as the internal loading control.

### 4.7. Apoptosis Protein Array and Plasmid Transfection

Apoptosis-related protein expression was analyzed using a human apoptosis proteome array. Survivin overexpression was achieved by plasmid transfection. SCC-1 and SCC-47 cells were seeded in 6-well plates at a density of approximately 2 × 10^5^ cells per well and cultured overnight. Cells were then transfected with a human survivin expression plasmid (Sino Biological, Beijing, China) using TurboFect transfection reagent (Thermo Fisher Scientific, Waltham, MA, USA) according to the manufacturer’s instructions. Briefly, 2 µg of plasmid DNA was diluted in 100 µL of serum-free medium and mixed with 4 µL TurboFect reagent. After incubation for 15–20 min at room temperature to allow complex formation, the mixture was added dropwise to the cells. Following 6 h of incubation, the medium was replaced with fresh complete medium, and the cells were cultured for an additional 24 h. Successful survivin overexpression was confirmed by Western blot analysis prior to jaceidin treatment. Cells transfected with an empty vector were used as the control group.

### 4.8. Statistical Analysis

All experiments were performed independently at least three times. Data are presented as mean ± SD. Statistical significance was determined by one-way ANOVA followed by Tukey’s post hoc test, as appropriate. For experiments involving jaceidin in combination with LY294002 or U0126, two-way ANOVA was performed to evaluate the main effects of jaceidin and the respective inhibitor, as well as their interaction. A *p* value < 0.05 was considered statistically significant.

## 5. Conclusions

In conclusion, we demonstrate that jaceidin decreased phosphorylation of AKT and ERK and modulated key apoptotic regulators by inhibiting Mcl-1 and increasing Bax, Bak and Bim. This further results in activation of caspase-3, -8, and -9 and PARP cleavage, reduced cell proliferation and apoptosis induction in OSCC. Although the present study demonstrates that jaceidin exerts significant anti-proliferative and pro-apoptotic effects in OSCC cells, several limitations should be acknowledged. In particular, the biosafety profile, pharmacokinetic properties, and ADME characteristics of jaceidin were not evaluated in the current study. These parameters are critical for assessing the translational potential of natural compounds as therapeutic agents. Therefore, further investigations, including in vivo toxicity evaluation, ADME prediction, and pharmacokinetic analyses, will be necessary to better determine the therapeutic feasibility of jaceidin for oral cancer treatment. However, further detailed studies are warranted.

## Data Availability

The original contributions presented in this study are included in the article. Further inquiries can be directed to the corresponding author.

## Abbreviations

The following abbreviations are used in this manuscript:

OSCC	Oral squamous cell carcinoma
PARP	Poly(ADP-ribose) polymerase
SCC	Squamous cell carcinoma
U0126	MEK1/2 inhibitor
LY294002	PI3K inhibitor
